# Inhibition of Fungal Pathogens across Genotypes and Temperatures by Amphibian Skin Bacteria

**DOI:** 10.3389/fmicb.2017.01551

**Published:** 2017-08-21

**Authors:** Carly R. Muletz-Wolz, Jose G. Almario, Samuel E. Barnett, Graziella V. DiRenzo, An Martel, Frank Pasmans, Kelly R. Zamudio, Luís Felipe Toledo, Karen R. Lips

**Affiliations:** ^1^Department of Biology, University of Maryland, College Park MD, United States; ^2^Center for Conservation Genomics, Smithsonian Conservation Biology Institute, National Zoological Park, Washington DC, United States; ^3^Department of Microbiology, Cornell University, Ithaca NY, United States; ^4^Department of Ecology, Evolution, and Marine Biology, University of California, Santa Barbara, Santa Barbara CA, United States; ^5^Department of Pathology, Bacteriology and Poultry Diseases, Ghent University Ghent, Belgium; ^6^Department of Ecology and Evolutionary Biology, Cornell University, Ithaca NY, United States; ^7^Department of Animal Biology, State University of Campinas Campinas, Brazil

**Keywords:** symbiont, salamander, *Batrachochytrium*, antifungal, disease ecology

## Abstract

Symbiotic bacteria may dampen the impacts of infectious diseases on hosts by inhibiting pathogen growth. However, our understanding of the generality of pathogen inhibition by different bacterial taxa across pathogen genotypes and environmental conditions is limited. Bacterial inhibitory properties are of particular interest for the amphibian-killing fungal pathogens (*Batrachochytrium dendrobatidis* and *Batrachochytrium salamandrivorans*), for which probiotic applications as conservation strategies have been proposed. We quantified the inhibition strength of five putatively *B. dendrobatidis*-inhibitory bacteria isolated from woodland salamander skin against six *Batrachochytrium* genotypes at two temperatures (12 and 18°C). We selected six genotypes from across the *Batrachochytrium* phylogeny: *B. salamandrivorans, B. dendrobatidis*-Brazil and four genotypes of the *B. dendrobatidis* Global Panzootic Lineage (GPL1: JEL647, JEL404; GPL2: SRS810, JEL423). We performed 96-well plate challenge assays in a full factorial design. We detected a *Batrachochytrium* genotype by temperature interaction on bacterial inhibition score for all bacteria, indicating that bacteria vary in ability to inhibit *Batrachochytrium* depending on pathogen genotype and temperature. *Acinetobacter rhizosphaerae* moderately inhibited *B. salamandrivorans* at both temperatures (μ = 46–53%), but not any *B. dendrobatidis* genotypes. *Chryseobacterium* sp. inhibited three *Batrachochytrium* genotypes at both temperatures (μ = 5–71%). *Pseudomonas* sp. strain 1 inhibited all *Batrachochytrium* genotypes at 12°C and four *Batrachochytrium* genotypes at 18°C (μ = 5–100%). *Pseudomonas* sp. strain 2 and *Stenotrophomonas* sp. moderately to strongly inhibited all six *Batrachochytrium* genotypes at both temperatures (μ = 57–100%). All bacteria consistently inhibited *B. salamandrivorans*. Using cluster analysis of inhibition scores, we found that more closely related *Batrachochytrium* genotypes grouped together, suggesting that bacterial inhibition strength may be predictable based on *Batrachochytrium* relatedness. We conclude that bacterial inhibition capabilities change among bacterial strains, *Batrachochytrium* genotypes and temperatures. A comprehensive understanding of bacterial inhibitory function, across pathogen genotypes and temperatures, is needed to better predict the role of bacterial symbionts in amphibian disease ecology. For targeted conservation applications, we recommend using bacterial strains identified as strongly inhibitory as they are most likely to produce broad-spectrum antimicrobial agents at a range of temperatures.

## Introduction

Interactions between host microbiomes and pathogens influence the severity of host disease. The outcome of microbiome-pathogen interactions can depend on microbiome composition (Chang et al., 2008; Jani and Briggs, 2014; Rovenich et al., 2014), pathogen genotype (Antwis et al., 2015), and environmental context (Duffy and Defago, 1999; Lokmer and Wegner, 2015). In amphibians, the skin microbiome has been implicated in variable host susceptibility to the disease chytridiomycosis (Harris et al., 2009a; Becker and Harris, 2010; Muletz et al., 2012). Chytridiomycosis has been linked to extreme loss of global amphibian biodiversity (Berger et al., 1998; Lips et al., 2006; Martel et al., 2014; Spitzen-van der Sluijs et al., 2016; Carvalho et al., 2017; Stegen et al., 2017), and is caused by skin infection by either of two congeneric chytrid fungi, *Batrachochytrium dendrobatidis* and *B. salamandrivorans*, hereafter *B. dendrobatidis* and *B. salamandrivorans*, respectively. One mechanism by which bacterial symbionts offer protection from *Batrachochytrium* is through production of inhibitory metabolites that can kill zoospores (Brucker et al., 2008a,b) or cause zoospores to move away from the metabolites (Lam et al., 2011). However, the impacts of amphibian skin microbiomes on fungal disease are difficult to predict because little is known about the ecological and evolutionary factors shaping microbiome functions, such as antifungal properties (e.g., Madison et al., 2017). To better predict the role of bacterial symbionts in amphibian disease ecology it is necessary to quantify antifungal properties across bacterial strains, *Batrachochytrium* genotypes and temperatures.

Application of antifungal bacteria has been proposed as a preventative strategy and a treatment option for chytridiomycosis in the wild (Muletz et al., 2012; Bletz et al., 2013). Of the approximately 250 bacterial operational taxonomic units (OTUs) identified as *B. dendrobatidis*-inhibitory (Woodhams et al., 2015; Muletz-Wolz et al., 2017a), nine have been used in bioaugmentation trials (e.g., Harris et al., 2009a,b; Woodhams et al., 2012; Nebergall, 2013; Becker et al., 2015a). These trials have had mixed success in mitigating chytridiomycosis. For instance, three studies found no effect of augmenting *Janthinobacterium lividum* on amphibian skin in reducing *B. dendrobatidis*-associated disease symptoms (Becker et al., 2011; Bletz, 2013; Nebergall, 2013), even though *J. lividum* inhibits *B. dendrobatidis* growth *in vitro* (Harris et al., 2006) and can be effective against *B. dendrobatidis* on amphibian skin (Harris et al., 2009a; Muletz et al., 2012). The variation in impact of bacterial augmentation *in vivo* suggests that environment and/or pathogen specific factors influence antifungal activity of bacterial symbionts through regulation of metabolite production or pathogen genotype specificity.

*Batrachochytrium* is a globally distributed genus with a complex evolutionary history within the two described species, *B. dendrobatidis* and *B. salamandrivorans* (Farrer et al., 2013, 2017; Rosenblum et al., 2013; Jenkinson et al., 2016). *B. dendrobatidis* originated at least 30 mya and is comprised of multiple, deeply diverged lineages including a Global Panzootic Lineage (GPL) and four enzootic lineages that are generally confined to their respective regions, *B. dendrobatidis*-Brazil, *B. dendrobatidis*-Cape, *B. dendrobatidis*-CH, and *B. dendrobatidis*-Korea (Schloegel et al., 2012; Farrer et al., 2013; Rosenblum et al., 2013; Martel et al., 2014; James et al., 2015; Jenkinson et al., 2016). Recent mass mortality events and population declines linked to *B. dendrobatidis* have been primarily associated with *B. dendrobatidis*-GPL (Fisher et al., 2009; Farrer et al., 2011; James et al., 2015). *B. dendrobatidis*-GPL is rapidly evolving with genetic differentiation that generally form two clades, *B. dendrobatidis*-GPL1, which is found primarily in North America, and *B. dendrobatidis*-GPL2, which is a geographically dispersed group (Schloegel et al., 2012; Rosenblum et al., 2013; James et al., 2015). *B. salamandrivorans* originated at least 30 mya where it coexisted with an Asian salamander clade (Martel et al., 2014) until its emergence in Western Europe resulted in rapid population declines of European fire salamanders (Martel et al., 2013; Stegen et al., 2017). To date, only *B. dendrobatidis*-GPL isolates have been tested to identify amphibian skin bacteria with *Batrachochytrium*-inhibitory traits (Antwis et al., 2015; Woodhams et al., 2015; Madison et al., 2017; Muletz-Wolz et al., 2017a).

Amphibian population declines and mass mortality events linked to *B. dendrobatidis* often have been more devastating in cooler seasons and higher elevations (Berger et al., 2004; Lips et al., 2006, 2008; Kriger and Hero, 2008; Carvalho et al., 2017). Host and pathogen responses may explain increased virulence at lower temperatures, including changes in (i) *B. dendrobatidis* fecundity as a life-history tradeoff (Woodhams et al., 2008), (ii) host immune response to infection (Ribas et al., 2009; Longo and Zamudio, 2017), and (iii) antifungal activity by bacterial symbionts (Daskin et al., 2014; Woodhams et al., 2014; Bresciano et al., 2015). For amphibian skin symbionts, temperature influences bacterial growth rate and population size, and high cell density is often needed to produce inhibitory metabolites (Yasumiba et al., 2016). Compared to *B. dendrobatidis* (Piotrowski et al., 2004; Stevenson et al., 2013), *B. salamandrivorans* generally has a lower optimal growth temperature (Martel et al., 2013) and *B. dendrobatidis*-inhibitory bacteria may not be effective against *B. salamandrivorans* due to temperature-dependent growth constraints of pathogen and bacteria, among other reasons [e.g., genetic and phenotypic variation between *B. dendrobatidis* and *B. salamandrivorans* (Farrer et al., 2017; Stegen et al., 2017)].

We quantified the inhibition strength of five amphibian skin bacteria cultured from North American woodland salamanders (*Plethodon cinereus* and *P. cylindraceus*), previously shown to inhibit *B. dendrobatidis* (GPL1-JEL404: Muletz-Wolz et al., 2017a) across *Batrachochytrium* genotypes and temperatures. We had three main objectives, (i) quantify the inhibitory proprieties of putatively anti-*B. dendrobatidis* bacterial strains against *B. salamandrivorans*, (ii) quantify the effect of temperature (12 and 18°C), *Batrachochytrium* genotype and their interaction on bacterial inhibition strength, and (iii) determine if *Batrachochytrium* relatedness predicts bacterial inhibition strength. Quantifying interactions between pathogen and bacterial symbionts in an environmental and genetic framework strengthens our understanding of disease dynamics and guides conservation measures.

## Materials and Methods

We performed *in vitro* challenge assays with five bacterial strains and six *Batrachochytrium* genotypes at two temperatures (12 and 18°C) using 96-well plates in a full factorial experimental design. We selected five bacterial strains to represent a range of inhibition based on their previously quantified inhibition strength against *B. dendrobatidis*-GPL1-JEL404 at 20°C (**Table 1**; Muletz-Wolz et al., 2017a). All bacterial strains were isolated from either *Plethodon cinereus* or *P. cylindraceus* at Shenandoah National Park, Virginia, in May 2012, and were widespread in these populations (Muletz-Wolz et al., 2017b). We selected six *Batrachochytrium* isolates from across the *Batrachochytrium* phylogeny, two GPL1 isolates (JEL647 and JEL404), two GPL2 isolates (SRS810 and JEL423), a Brazilian *B. dendrobatidis* isolate (JEL649), and *B. salamandrivorans* (**Table 2**). Hereafter, we refer to these *Batrachochytrium* isolates as *Batrachochytrium* genotypes because genetic analyses have shown that each of these isolates represent distinct genotypes (Schloegel et al., 2012; Martel et al., 2013; James et al., 2015).

**Table 1:** Five bacteria strains used in the study, including their phylogenetic designation and previously quantified inhibition strength against *B. dendrobatidis*-GPL1-JEL404 (Muletz-Wolz et al., 2017a).

Bacteria strain	Strain ID	GenBank accession no. of 16S rRNA gene	Inhibition score for *B. dendrobatidis*-GPL1-JEL404 at 20°C (%)
*Acinetobacter rhizosphaerae*	THA6-B68	KU739019	32
*Pseudomonas* sp. strain 1	RSB5-4	KU738948	99
*Pseudomonas* sp. strain 2	SFB8-6	KU738987	82
*Chryseobacterium* sp.	SFA2-10	KU738960	54
*Stenotrophomonas* sp.	LSB7-4	KU738931	100

**Table 2:** Six *Batrachochytrium* genotypes used in the study.

Isolate ID	Phylogenetic lineage	Genotype	Location of isolation	Approximately # passages since isolation
JEL649	*B. dendrobatidis*-Brazil	Brazil-JEL649	São Paulo, Brazil	8
JEL647	*B. dendrobatidis*-GPL1	GPL1-JEL647	California, United States	3
SRS810	*B. dendrobatidis*-GPL2	GPL2-SRS810	Georgia, United States	6
AMFP13/1	*B. salamandrivorans*	*B. salamandrivorans*	Zuid-Limburg, Netherlands	9
JEL423	*B. dendrobatidis*-GPL2	GPL2-JEL423	El Cope, Panama	6
JEL404	*B. dendrobatidis*-GPL1	GPL1-JEL404	Maine, United States	8

*Each isolate represented a distinct genotype based on genetic analyses by Schloegel et al. (2012), Martel et al. (2013), and James et al. (2015). We used the combination of *Batrachochytrium* lineage and isolate to specify the *Batrachochytrium* genotype.*

### Experimental Set-up

We performed the experiment using a total of 16 96-well plates. Each plate was assigned to one of four randomly generated configurations of bacterial by *Batrachochytrium* combinations (Supplementary Figure S1), and housed in one of four incubators (Percival model DR-36VL; two chambers per temperature, Supplementary Figure S2). Each incubator contained a total of four plates, with one plate per configuration.

We set up challenge assays following a protocol based on Muletz-Wolz et al. (2017a), with the following modifications to accommodate the design of the experiment. We passaged cryopreserved bacteria on 1% tryptone plates three times, then inoculated each bacterial strain in 25 mL of 1% tryptone broth and grew for 3 days at room temperature (approximately 21°C) on a shaker at 100 rpm. By 3 days of incubation, the bacterial cultures reached high densities where inhibitory metabolites are produced (Bérdy, 2005). We obtained cell-free supernatants (CFSs) from bacterial monocultures, following the centrifuging and filtering methods outlined in Muletz-Wolz et al. (2017a). By using bacterial CFS, we determined the inhibitory properties of bacterially produced extracellular factors against live *Batrachochytrium* zoospores, and eliminated the possibility of direct competition or priority effects between bacterial and fungal cultures. As the bacterial strains were grown at one temperature prior to the experiment all effects of temperature on inhibition relate to temperature-dependent activity of CFS extracellular factors and/or fungal physiology. For *Batrachochytrium* genotypes, we passaged cryopreserved isolates (prior passage history ranged between 3 and 9 times; **Table 2**) on 1% tryptone plates twice, and then grew them for 1 week on multiple 1% tryptone plates at 15°C. We harvested zoospores by flooding the plates with 1% tryptone broth, filtered out the zoosporangia using a sterile coffee filter, and homogenized the zoospore mixture.

To set up the assays, we added 50 μl of approximately 1 × 10^6^ zoospores/ml of each *Batrachochytrium* genotype (counted with a hemocytometer; approximately 50,000 zoospores in each well) to their designated wells in a 96-well plate (Supplementary Figure S1). In sample wells, we added 50 μl of the CFS from each bacterial strain to four wells for each bacterial-*Batrachochytrium* combination. In total, each bacterial-*Batrachochytrium* combination was represented in 16 wells distributed over four plates per temperature. In each 96-well assay, we included two positive controls (PCs) and one negative control for each *Batrachochytrium* genotype using four wells per control. The positive controls were: 50 μl of *Batrachochytrium* zoospores + 50 μl 1% tryptone broth PC and 50 μl of *Batrachochytrium* zoospores + 50 μl of water [nutrient-depleted positive control (NDPC)]. The negative control was 50 μl of *Batrachochytrium* zoospores heat-killed at 60°C for 60 min + 50 μl of 1% tryptone broth (heat-killed *B. dendrobatidis*: HK). We measured optical density (OD_492 nm_) of each well for 16 96-well plates using a microplate reader every other day starting on day 1, for 27 days.

### *B. dendrobatidis* Inhibition Score Calculations

We used R version 3.2.5 for all calculations and statistical analyses (R Core Team, 2016). We visually inspected the optical density (OD) readings for each plate, and excluded data points for 32 wells (2% of wells) with unusually high densities (+0.1 or greater well OD compared to replicate wells on same plate), indicating contamination or error. We corrected for baseline zoospore OD by subtracting the average heat-killed OD of each *Batrachochytrium* genotype from the corresponding experimental wells in each plate. To achieve a normal distribution, we transformed the corrected OD readings using the following equation, log(OD_corrected_(1-OD_corrected_)+1). Next, we fit linear regressions to the transformed OD readings over time for each well with the intercept set at zero. We extracted the slope of the linear regression, and interpreted this as *Batrachochytrium* growth (i.e., Δ optical density/time). We excluded wells in which the linear model had an r^2^ less than 0.20, given the poor fit of the data. After quality filtering, we had slopes for 867 of the 960 sample wells (Supplementary Table S1). Then, we calculated *Batrachochytrium* growth inhibition, hereafter referred to as inhibition score, by dividing the slope of each sample well by the slope of the average NDPC wells of the corresponding *Batrachochytrium* genotype on the same plate, and subtracting the subsequent fraction from one, [Inhibition score = 1 – (slope sample well/average slope NDPC)]. We compared the slopes of sample wells to the NDPC wells because this accounts for the issue of nutrient depletion in PC wells and is a more conservative approach in identifying anti-*Batrachochytrium* bacteria (Bell et al., 2013; Muletz-Wolz et al., 2017a). We created this standardized inhibition scoring system to be able to compare inhibition scores between the two experimental temperatures. We interpreted inhibition scores greater than zero as inhibitory, indicating that the bacterial-*Batrachochytrium* sample well had less growth than the NDPC wells. Values less than zero we interpreted as non-inhibitory, and to determine if any bacterial symbionts promoted *Batrachochytrium* growth, we compared the inhibition scores to those of the PC wells (see Statistical Analyses below; Supplementary Figure S3).

### Statistical Analyses

We quantified the effects of *Batrachochytrium* genotype, temperature, and their interaction (explanatory variables) on inhibition scores (response variable) using a linear mixed-effects model for each bacterial strain separately, using the *lmer* function in the ‘lme4’ package (Bates et al., 2015). We included plate nested within incubator as a random effect in each model. Next, we used the *Anova* function in the ‘car’ package with type II sum of squares to determine the significance of each of the fixed-effects (Fox and Weisberg, 2011). Using the ‘lsmeans’ package (Lenth, 2016), we used the *lsmeans* function to perform *post hoc* analyses to determine significant difference among *Batrachochytrium* genotypes and between temperatures. We used the *lsmip* function to generate *Batrachochytrium* genotypes by temperature interaction plots for each bacterial strain.

We determined if any bacterial symbiont promoted *Batrachochytrium* growth by comparing the inhibition score of each bacterial *Batrachochytrium* combination at each temperature to the inhibition score of the PC well for the corresponding *Batrachochytrium* genotype (Supplementary Figure S3). We used a linear mixed-effects model for each *Batrachochytrium* genotype examining the effects of well type (i.e., bacteria or PC), temperature, and their interaction (explanatory variables) on inhibition scores (response variable). We included the same random effects, determined significance and conducted *post hoc* analyses as described above.

We determined if patterns of bacterial inhibition score reflected *Batrachochytrium* phylogenetic relatedness by conducting a cluster analysis using the mean inhibition score for each bacteria-*Batrachochytrium* combination at each temperature. We used two clustering methods to confirm similar clustering patterns: (i) Ward’s hierarchical clustering with Euclidean distances using the *pvclust* function in the ‘pvclust’ package (Suzuki and Shimodaira, 2006), and (ii) k-means clustering using a plot of within groups sum of squares by number of clusters to determine the appropriate number of clusters (Everitt and Hothorn, 2009). We were unable to perform a full phylogenetic analysis because we could not calculate branch length due to missing genotype data for *B. salamandrivorans* and GPL1-JEL404 (Schloegel et al., 2012, T. James, pers. comm.).

## Results

*Batrachochytrium salamandrivorans* was the only *Batrachochytrium* genotype that was inhibited by all bacterial strains, with moderate to strong inhibition (μ = 43–92%) at both temperatures. *Stenotrophomonas* sp. and *Pseudomonas* sp. strain 2 were the most inhibitory against *B. salamandrivorans* at both temperatures (μ = 91–92%).

We detected a *Batrachochytrium* genotype by temperature interaction effect on inhibition score for all bacterial strains (*post hoc* analyses: Supplementary Tables S2, S3), indicating that bacterial inhibition strength is affected by both *Batrachochytrium* genotype and temperature. No bacterial strain promoted the growth of any *Batrachochytrium* genotype (Supplementary Figure S3).

**FIGURE 1:**
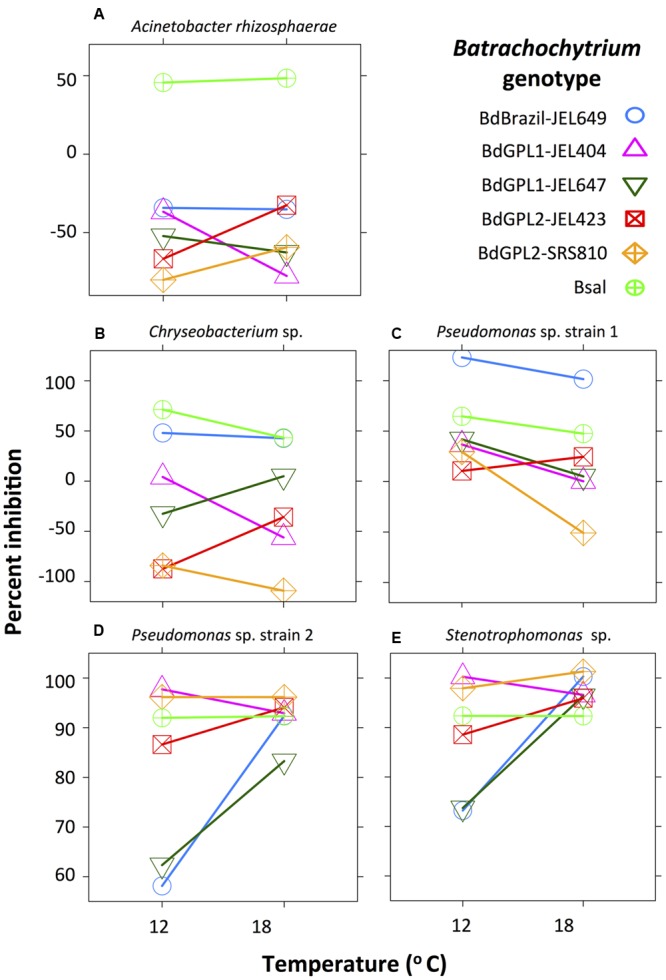
Interaction plot showing the effect of *Batrachochytrium* genotype and temperature on bacterial inhibition score (percent inhibition: note different scale on y-axis). *Acinetobacter rhizosphaerae* **(A)** was moderately inhibitory of *B. salamandrivorans* at both temperatures, but did not inhibit any *B. dendrobatidis* genotypes. *Chryseobacterium* sp. **(B)** and *Pseudomonas* sp. strain 1 **(C)** were weakly to strongly inhibitory of most *Batrachochytrium* genotypes. *Pseudomonas* sp. strain 2 **(D)** and *Stenotrophomonas* sp. **(E)** were moderately to strongly inhibitory of all *Batrachochytrium* genotypes at both temperatures, and showed similar inhibition patterns. Percent inhibition is based on the parameter estimates from the linear-mixed effects models.

*Acinetobacter rhizosphaerae* only inhibited *B. salamandrivorans* (**Figure 1A**), and was moderately inhibitory of *B. salamandrivorans* at both temperatures (μ = 46–53%). Inhibition scores for *A. rhizosphaerae* differed among pathogen genotypes (*X*^2^ = 202.7, df = 5, *p* < 0.001) and this depended on temperature (interaction term: *X*^2^ = 25.3, df = 5, *p* < 0.001), but the significant interaction was for scores that were non-inhibitory (**Figure 1A**).

Two bacterial strains, *Chryseobacterium* sp. and *Pseudomonas* sp. strain 1, were weakly to strongly inhibitory of most *Batrachochytrium* genotypes. Inhibition scores for *Chryseobacterium* sp. differed among *Batrachochytrium* genotypes (*X*^2^ = 932.9, df = 5, *p* < 0.001) and this depended on temperature (interaction term: *X*^2^ = 122.0, df = 5, *p* < 0.001). *Chryseobacterium* sp. was moderately inhibitory of *B. salamandrivorans* and Brazil-JEL649 at both temperatures (μ = 38–71%), and was weakly inhibitory of the GPL1 genotypes with GPL1-JEL647 only inhibited at 18°C and GPL1-JEL404 only inhibited at 12°C (**Figure 1B**). Inhibition scores for *Pseudomonas* sp. strain 1 differed among genotypes (*X*^2^ = 479.1, df = 5, *p* < 0.001), temperatures (*X*^2^ = 22.8, df = 1, *p* < 0.001) and showed a significant interaction (*X*^2^ = 57.4, df = 5, *p* < 0.001). *Pseudomonas* sp. strain 1 inhibited all *Batrachochytrium* genotypes (μ = 5–100%), except for GPL1-JEL404 and GPL2-SRS810 at 18°C (**Figure 1C**). *Pseudomonas* sp. strain 1 was significantly more inhibitory of GPL1-JEL404, GPL1-JEL647, GPL2-SRS810 at 12°C compared to 18°C, and was differentially inhibitory among *Batrachochytrium* genotypes (Supplementary Table S3). For instance, *Pseudomonas* sp. strain 1 was more inhibitory of Brazil-JEL649 at both temperatures compared to all other *Batrachochytrium* genotypes.

Two bacterial strains, *Pseudomonas* sp. strain 2 and *Stenotrophomonas* sp., were inhibitory of all *Batrachochytrium* genotypes at both temperatures. Inhibition scores for *Pseudomonas* sp. strain 2 differed among genotypes (*X*^2^ = 219.2, df = 5, *p* < 0.001), temperatures (*X*^2^ = 10.4, df = 1, *p* = 0.001) and showed a significant interaction (*X*^2^ = 101.1, df = 5, *p* < 0.001). *Pseudomonas* sp. strain 2 moderately to strongly inhibited all *Batrachochytrium* genotypes at both temperatures (μ = 57–98%). Inhibition scores for *Stenotrophomonas* sp. differed among *Batrachochytrium* genotypes (*X*^2^ = 40.6, df = 5, *p* < 0.001), temperatures (*X*^2^ = 5.5, df = 1, *p* = 0.02) and showed a significant interaction (*X*^2^ = 31.9, df = 5, *p* < 0.001). *Stenotrophomonas* sp. strongly inhibited all *Batrachochytrium* genotypes at both temperatures (μ = 70–100%), but was less inhibitory of GPL1-JEL647 and Brazil-JEL649 at 12°C than all other genotypes, except for GPL2-JEL423 (**Figure 1D** and Supplementary Table S3). Similar to *Pseudomonas* sp. strain 2, *Stenotrophomonas* sp. was less inhibitory of GPL1-JEL647 and Brazil-JEL649 at 12°C than all other *Batrachochytrium* genotypes (**Figure 1E** and Supplementary Table S3).

**FIGURE 2:**
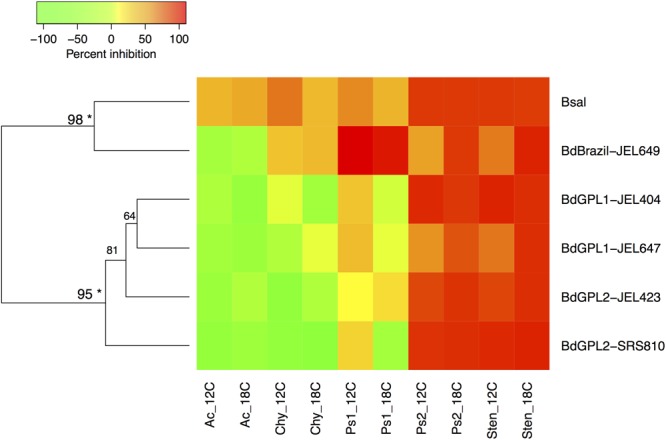
Heatmap displaying Ward’s hierarchical clustering of *Batrachochytrium* genotypes based on mean bacterial inhibition scores at each temperature. Approximately unbiased *p*-values are indicated for each cluster, with values larger than 95% being strongly supported by the data (^∗^). Two clusters were supported by the data, one including *B. dendrobatidis*-Brazil and *B. salamandrivorans*, and one clustering all *B. dendrobatidis*-GPL genotypes. Ac = *Acinetobacter rhizosphaerae*, Chy = *Chryseobacterium* sp., Ps1 = *Pseudomonas* sp. strain 1, Ps2 = *Pseudomonas* sp. strain 2, Sten = *Stenotrophomonas* sp.

Bacterial inhibition strength may be predictable based on *Batrachochytrium* phylogenetic relatedness. Both clustering methods supported the same two clusters within the data, with *B. dendrobatidis*-Brazil and *B. salamandrivorans* clustering together and the *B. dendrobatidis*-GPL genotypes forming a separate cluster (**Figure 2**).

## Discussion

Our findings suggest that many bacteria identified as *Batrachochytrium*-inhibitory *in vitro* are unlikely to be consistently effective in mitigation of chytridiomycosis *in vivo*. We found that all bacterial strains consistently inhibited *B. salamandrivorans* growth across temperatures (12 and 18°C), but that three of the five bacterial strains did not consistently inhibit the five *B. dendrobatidis* genotypes. Similarly, Antwis et al. (2015) found that 3 of 11 previously identified *B. dendrobatidis*-inhibitory bacterial strains did not consistently inhibit three *B. dendrobatidis* isolates at 18°C. These results highlight the importance of considering *Batrachochytrium* genotype and bacterial strain in host–pathogen interactions, and may explain the variation in effectiveness of probiotics in experimental trials (e.g., Harris et al., 2009a; Woodhams et al., 2012; Becker et al., 2015a).

Our results also highlight the importance of considering environmental context in host–pathogen interactions. The effect of temperature on inhibition was not unidirectional, but depended on the context of the interacting *Batrachochytrium* genotype and bacterial strain. For example, *Pseudomonas* sp. strain 1 was less inhibitory of GPL1-JEL647 at 12°C compared to 18°C, whereas *Pseudomonas* sp. strain 2 and *Stenotrophomonas* sp. were more inhibitory of GPL1-JEL647 at 12°C compared to 18°C. The differences in inhibition between temperatures of the same *Batrachochytrium* genotype likely related to temperature-dependent changes in bacterial extracellular factor activity (Daskin et al., 2014) and/or *Batrachochytrium* physiology (Woodhams et al., 2008). Bacterially produced extracellular factors include bacteriocins, siderophores, organic acids, lysozymes, proteases, and secondary metabolites. However, the most likely factors contributing to inhibition are secondary metabolites (reviewed by Verschuere et al., 2000; Raaijmakers et al., 2002), and antimicrobial activity of metabolites can vary among temperatures (Humair et al., 2009; Aguilar and Klotz, 2010). In addition, *Batrachochytrium* isolates vary in phenotypic traits (e.g., growth rate and zoosporangium size) depending on temperature (Piotrowski et al., 2004; Martel et al., 2013; Stevenson et al., 2013) and this may impact their susceptibility to inhibitory compounds. Investigation into the inhibition strength of *Batrachochytrium*-inhibitory bacterial metabolites (e.g., violacein) at different temperatures would provide insight into whether bacterial factors or *Batrachochytrium* physiology underpin the temperature-dependent differences in inhibition.

Our study supports the hypothesis that similarity of fungal genomes can predict strength of bacterial inhibition. Our results showed separation between two clusters: the two highly divergent lineages (*B. salamandrivorans* and *B. dendrobatidis*-Brazil) and the GPL genotypes. Generally, *B. salamandrivorans* and *B. dendrobatidis*-Brazil were more likely to be inhibited than the GPL genotypes. The GPL genotypes have increased chromosomal copy numbers (CCNs) and loss of heterozygosity compared to the endemic *B. dendrobatidis* lineages, and these measures are associated with increased virulence (Schloegel et al., 2012; Farrer et al., 2013; Rosenblum et al., 2013; James et al., 2015; Becker et al., 2017). This association may relate to resistance to inhibitory compounds released by host or symbionts. For instance, Farrer et al. (2013) found that *B. dendrobatidis* CCN increased following exposure to a host-produced antimicrobial peptide, which then resulted in reduced inhibition. A greater sampling across *Batrachochytrium* genotypes would be a useful next step to confirm this lineage-based similarity in pathogen response to bacterial inhibition.

While there was a relationship between *Batrachochytrium* relatedness and inhibition patterns, we did not detect a relationship between bacterial relatedness and inhibition patterns. For instance, we found variation within the two *Pseudomonas* sp. strains tested, which were defined as the same OTU (16S rRNA sequence similarity = 99%). *Pseudomonas* sp. strain 1 was generally less inhibitory compared to *Pseudomonas* sp. strain 2, and also non-inhibitory of two pathogen genotypes at 18°C. These findings are similar to other studies that profiled larger numbers of bacterial strains in a phylogenetic framework and found that inhibition strength was not correlated to bacterial phylogeny (Becker et al., 2015b; Muletz-Wolz et al., 2017a). While the majority of genomic information can be highly similar within a bacterial OTU, gene clusters associated with inhibitory metabolite production can differ among strains (Chen et al., 2015), potentially explaining the discrepancy in inhibition strength within an OTU. On the other hand, different OTUs can show similar inhibition patterns as homologous recombination and horizontal gene transfer of genes encoding antifungal compounds can occur between bacterial lineages with levels of DNA divergence as high as 25% (Kinashi et al., 1987; Ravel et al., 2000; Cohan, 2001). For example, we found a strong similarity in the inhibition patterns of *Pseudomonas* sp. strain 2 and *Stenotrophomonas* sp. (16S rRNA sequence similarity = 75%). Documented modes of antibiosis by *Pseudomonas* differ from those of *Stenotrophomonas* spp. (Raaijmakers et al., 2002; Compant et al., 2005); whole genome sequencing or chemical analyses of these bacterial strains may identify the specific agent(s) underlying this similarity in *Batrachochytrium* inhibition.

For targeted conservation applications, we suggest testing bacterial strains *in vitro* against multiple *Batrachochytrium* genotypes across a range of temperatures to identify probiotics that would be most effective at mitigating *Batrachochytrium* infection *in situ*. If probiotic-based conservation strategies are needed rapidly, previously identified *Batrachochytrium*-inhibitory bacterial strains that are strongly inhibitory are the most likely to be effective. For instance, we found that two bacterial strains (*Pseudomonas* sp. strain 2 and *Stenotrophomonas* sp.) were strongly inhibitory of all *Batrachochytrium* genotype tested across temperatures. These bacterial strains are good probiotic candidates as they likely produce antifungal compounds at a range of temperatures that inhibit a range of pathogen genotypes (Bletz et al., 2013).

## Conclusion

We quantified the inhibition of diverse *Batrachochytrium* genotypes by five bacterial strains that were isolated from woodland salamanders in the eastern United States and previously shown to inhibit an eastern US *B. dendrobatidis* genotype, GPL1-JEL404 (Muletz-Wolz et al., 2017a). Inhibition capabilities of the symbiotic bacteria changed as a function of bacterial strain, *Batrachochytrium* genotype and temperature. This has practical implications for understanding host–pathogen dynamics and developing conservation measures. Amphibians and their microbiomes will continue to be exposed to rapidly evolving *Batrachochytrium* genotypes, and hosts harboring higher numbers of microbial taxa and hence more potentially inhibitory species may provide greater resistance to pathogen invasion than microbiome communities with fewer taxa (Chang et al., 2008; Ling et al., 2015; Lokmer and Wegner, 2015; Longo et al., 2015). Our findings should also be considered in other systems, such as white-nose syndrome in bats and pathogens in agriculture, where probiotic application is used as a disease management strategy (Verschuere et al., 2000; Raaijmakers et al., 2002; Teplitski and Ritchie, 2009; Hoyt et al., 2015; Xue et al., 2015).

## Data Accessibility

All experimental data and statistical analyses (R code) will be deposited upon acceptance in figshare (doi: 10.6084/m9.figshare.5297416).

## Author Contributions

CM-W, GD, KZ, LT, and KL designed the research. AM, FP, KZ, and LT provided the *Batrachochytrium* isolates. CM-W, JA, and SB conducted the lab work. CM-W and JA analyzed the data with advice from SB and GD. All authors contributed to the interpretation of the data. CM-W wrote the manuscript and all authors provided critical feedback. All authors approved the manuscript’s content.

## Conflict of Interest Statement

The authors declare that the research was conducted in the absence of any commercial or financial relationships that could be construed as a potential conflict of interest.

## References

[B1] Aguilar C. Klotz B. (2010). Effect of the temperature on the antagonistic activity of lactic acid bacteria against *Escherichia coli* and *Listeria monocytogenes*. *J. Food Saf.* 30 996–1015. 10.1111/j.1745-4565.2010.00257.x

[B2] Antwis R. E. Preziosi R. F. Harrison X. A. Garner T. W. (2015). Amphibian symbiotic bacteria do not show a universal ability to inhibit growth of the global panzootic lineage of *Batrachochytrium dendrobatidis*. *Appl. Environ. Microbiol.* 81 3706–3711. 10.1128/Aem.00010-15 25819964 PMC4421062

[B3] Bates D. Mächler M. Bolker B. Walker S. (2015). Fitting linear mixed-effects models using lme4. *J. Stat. Softw.* 67 1–47. 10.18637/jss.v067.i01

[B4] Becker C. G. Greenspan S. E. Tracy K. E. Dash J. A. Lambertini C. Jenkinson T. S. (2017). Variation in phenotype and virulence among enzootic and panzootic amphibian chytrid lineages. *Fungal Ecol.* 26 45–50.

[B5] Becker M. H. Harris R. N. (2010). Cutaneous bacteria of the redback salamander prevent morbidity associated with a lethal disease. *PLoS ONE* 5:e10957 10.1371/journal.pone.0010957 PMC2881031 20532032

[B6] Becker M. H. Harris R. N. Minbiole K. P. C. Schwantes C. R. Rollins-Smith L. A. Reinert L. K. (2011). Towards a better understanding of the use of probiotics for preventing chytridiomycosis in Panamanian golden frogs. *Ecohealth* 8 501–506. 10.1007/s10393-012-0743-0 22328095

[B7] Becker M. H. Walke J. B. Cikanek S. Savage A. E. Mattheus N. Santiago C. N. (2015a). Composition of symbiotic bacteria predicts survival in Panamanian golden frogs infected with a lethal fungus. *Proc. Biol. Sci.* 282:20142881 10.1098/rspb.2014.2881 PMC4389611 25788591

[B8] Becker M. H. Walke J. B. Murrill L. Woodhams D. C. Reinert L. K. Rollins-Smith L. A. (2015b). Phylogenetic distribution of symbiotic bacteria from Panamanian amphibians that inhibit growth of the lethal fungal pathogen *Batrachochytrium dendrobatidis*. *Mol. Ecol.* 24 1628–1641. 10.1111/mec.13135 25737297

[B9] Bell S. C. Alford R. A. Garland S. Padilla G. Thomas A. D. (2013). Screening bacterial metabolites for inhibitory effects against *Batrachochytrium dendrobatidis* using a spectrophotometric assay. *Dis. Aquat. Organ.* 103 77–85. 10.3354/dao02560 23482387

[B10] Bérdy J. (2005). Bioactive microbial metabolites - a personal view. *J. Antibiot.* 58 1–26. 10.1038/ja.2005.1 15813176

[B11] Berger L. Speare R. Daszak P. Green D. E. Cunningham A. A. Goggin C. L. (1998). Chytridiomycosis causes amphibian mortality associated with population declines in the rain forests of Australia and Central America. *Proc. Natl. Acad. Sci. U.S.A.* 95 9031–9036. 10.1073/pnas.95.15.903 9671799 PMC21197

[B12] Berger L. Speare R. Hines H. B. Marantelli G. Hyatt A. D. McDonald K. R. (2004). Effect of season and temperature on mortality in amphibians due to chytridiomycosis. *Aust. Vet. J.* 82 434–439. 10.1111/j.1751-0813.2004.tb11137.x 15354853

[B13] Bletz M. (2013). *Probiotic Bioaugmentation of an Anti-Bd Bacteria, Janthinobacterium lividum, on the Amphibian, Notophthalmus viridescens, Transmission Efficacy and Persistence of the Probiotic on the Host and Non-Target Effects of Probiotic Addition on Ecosystem Components.* Master’s thesis, James Madison University Harrisonburg, VA.

[B14] Bletz M. C. Loudon A. H. Becker M. H. Bell S. C. Woodhams D. C. Minbiole K. P. C. (2013). Mitigating amphibian chytridiomycosis with bioaugmentation, characteristics of effective probiotics and strategies for their selection and use. *Ecol. Lett.* 16 807–820. 10.1111/ele.12099 23452227

[B15] Bresciano J. C. Salvador C. A. Paz-y-Mino C. Parody-Merino A. M. Bosch J. Woodhams D. C. (2015). Variation in the presence of anti-*Batrachochytrium dendrobatidis* bacteria of amphibians across life stages and elevations in Ecuador. *Ecohealth* 12 310–319. 10.1007/s10393-015-1010-y 25669915

[B16] Brucker R. M. Baylor C. M. Walters R. L. Lauer A. Harris R. N. Minbiole K. P. C. (2008a). The identification of 2,4-diacetylphloroglucinol as an antifungal metabolite produced by cutaneous bacteria of the salamander *Plethodon cinereus*. *J. Chem. Ecol.* 34 39–43. 10.1007/s10886-007-9352-8 18058176

[B17] Brucker R. M. Harris R. N. Schwantes C. R. Gallaher T. N. Flaherty D. C. Lam B. A. (2008b). Amphibian chemical defense, Antifungal metabolites of the microsymbiont *Janthinobacterium lividum* on the salamander *Plethodon cinereus*. *J. Chem. Ecol.* 34 1422–1429. 10.1007/s10886-008-9555-7 18949519

[B18] Carvalho T. Becker C. G. Toledo L. F. (2017). Historical amphibian declines and extinctions in Brazil linked to chytridiomycosis. *Proc. Biol. Sci.* 284:20162254 10.1098/rspb.2016.2254 PMC5310605 28179514

[B19] Chang J. Y. Antonopoulos D. A. Kalra A. Tonelli A. Khalife W. T. Schmidt T. M. (2008). Decreased diversity of the fecal microbiome in recurrent Clostridium difficile-associated diarrhea. *J. Infect. Dis.* 197 435–438. 10.1086/525047 18199029

[B20] Chen Y. W. Shen X. M. Peng H. S. Hu H. B. Wang W. Zhang X. H. (2015). Comparative genomic analysis and phenazine production of *Pseudomonas chlororaphis*, a plant growth-promoting rhizobacterium. *Genomics Data* 4 33–42. 10.1016/j.gdata.2015.01.006 26484173 PMC4535895

[B21] Cohan F. M. (2001). Bacterial species and speciation. *Syst. Biol.* 50 513–524. 10.1080/10635150118398 12116650

[B22] Compant S. Duffy B. Nowak J. Clement C. Barka E. A. (2005). Use of plant growth-promoting bacteria for biocontrol of plant diseases, Principles, mechanisms of action, and future prospects. *Appl. Environ. Microbiol.* 71 4951–4959. 10.1128/aem.71.9.4951-4959.2005 16151072 PMC1214602

[B23] Daskin J. H. Bell S. C. Schwarzkopf L. Alford R. A. (2014). Cool temperatures reduce antifungal activity of symbiotic bacteria of threatened amphibians - implications for disease management and patterns of decline. *PLoS ONE* 9:e100378 10.1371/journal.pone.0100378 PMC4062522 24941262

[B24] Duffy B. K. Defago G. (1999). Environmental factors modulating antibiotic and siderophore biosynthesis by *Pseudomonas fluorescens* biocontrol strains. *Appl. Environ. Microbiol.* 65 2429–2438. 10347023 10.1128/aem.65.6.2429-2438.1999 PMC91358

[B25] Everitt B.S. Hothorn T. (2009). *A Handbook of Statistical Analyses Using R* 2nd Edn. Boca Raton, Fl: CRC Press.

[B26] Farrer R. A. Henk D. A. Garner T. W. J. Balloux F. Woodhams D. C. Fisher M. C. (2013). Chromosomal copy number variation, selection and uneven rates of recombination reveal cryptic genome diversity linked to pathogenicity. *PLoS Genet.* 9:e1003703 10.1371/journal.pgen.1003703 PMC3744429 23966879

[B27] Farrer R. A. Martel A. Verbrugghe E. Abouelleil A. Ducatelle R. Longcore J. E. (2017). Genomic innovations linked to infection strategies across emerging pathogenic chytrid fungi. *Nat.* *Commun.* 8:14742. 10.1038/ncomms14742 PMC5364385 28322291

[B28] Farrer R. A. Weinert L. A. Bielby J. Garner T. W. J. Balloux F. Clare F. (2011). Multiple emergences of genetically diverse amphibian-infecting chytrids include a globalized hypervirulent recombinant lineage. *Proc. Natl. Acad. Sci. U.S.A.* 108 18732–18736. 10.1073/pnas.1111915108 22065772 PMC3219125

[B29] Fisher M. C. Garner T. W. J. Walker S. F. (2009). Global emergence of *Batrachochytrium dendrobatidis* and amphibian chytridiomycosis in space, time, and host. *Annu. Rev. Microbiol.* 63 291–310. 10.1146/annurev.micro.091208.073435 19575560

[B30] Fox J. Weisberg S. (2011). *An R Companion to Applied Regression* Second Edn. Thousand Oaks, CA: Sage.

[B31] Harris R. N. Brucker R. M. Walke J. B. Becker M. H. Schwantes C. R. Flaherty D. C. (2009a). Skin microbes on frogs prevent morbidity and mortality caused by a lethal skin fungus. *Isme J.* 3 818–824. 10.1038/ismej.2009.27 19322245

[B32] Harris R. N. Lauer A. Simon M. A. Banning J. L. Alford R. A. (2009b). Addition of antifungal skin bacteria to salamanders ameliorates the effects of chytridiomycosis. *Dis. Aquat. Organ.* 83 11–16. 10.3354/dao02004 19301631

[B33] Harris R. N. James T. Y. Lauer A. Simon M. A. Patel A. (2006). Amphibian pathogen *Batrachochytrium dendrobatidis* is inhibited by the cutaneous bacteria of amphibian species. *EcoHealth* 3 53–56. 10.1007/s10393-005-0009-1

[B34] Hoyt J. R. Cheng T. L. Langwig K. E. Hee M. M. Frick W. F. Kilpatrick A. M. (2015). Bacteria isolated from bats inhibit the growth of *Pseudogymnoascus destructans*, the causative agent of White-Nose Syndrome. *PLoS ONE* 10:e0121329 10.1371/journal.pone.0121329 PMC4390377 25853558

[B35] Humair B. Gonzalez N. Mossialos D. Reimmann C. Haas D. (2009). Temperature-responsive sensing regulates biocontrol factor expression in *Pseudomonas fluorescens* Cha0. *Isme J.* 3 955–965. 10.1038/ismej.2009.42 19421236

[B36] James T. Y. Toledo L. F. Rodder D. Leite D. D. Belasen A. M. Betancourt-Roman C. M. (2015). Disentangling host, pathogen, and environmental determinants of a recently emerged wildlife disease, lessons from the first 15years of amphibian chytridiomycosis research. *Ecol. Evol.* 5 4079–4097. 10.1002/ece3.1672 26445660 PMC4588650

[B37] Jani A. J. Briggs C. J. (2014). The pathogen *Batrachochytrium dendrobatidis* disturbs the frog skin microbiome during a natural epidemic and experimental infection. *Proc. Natl. Acad. Sci. U.S.A.* 111 E5049–E5058. 10.1073/pnas.1412752111 25385615 PMC4250152

[B38] Jenkinson T. S. Betancourt-Román C. M. Lambertini C. Valencia-Aguilar A. Rodriguez D. Nunes-de-Almeida C. H. L. (2016). Amphibian-killing chytrid in Brazil comprises both locally endemic and globally expanding populations. *Mol. Ecol.* 25 2978–2996. 10.1111/mec.13599 26939017

[B39] Kinashi H. Shimaji M. Sakai A. (1987). Giant linear plasmids in Streptomyces which code for antibiotic biosysthesis genes. *Nature* 328 454–456. 10.1038/328454a0 3614348

[B40] Kriger K. M. Hero J. -M. (2008). Altitudinal distribution of chytrid (*Batrachochytrium dendrobatidis*) infection in subtropical Australian frogs. *Austral Ecol.* 33 1022–1032. 10.1111/j.1442-9993.2008.01872.x

[B41] Lam B. A. Walton D. B. Harris R. N. (2011). Motile zoospores of *Batrachochytrium dendrobatidis* move away from antifungal metabolites produced by amphibian skin bacteria. *Ecohealth* 8 36–45. 10.1007/s10393-011-0689-7 21769695

[B42] Lenth R. (2016). *lsmeans, Least-Squares Means. R Package Version* 2. 20–23. Available at: http://CRAN.R-project.org/package=lsmeans

[B43] Ling Z. Liu X. Cheng Y. Jiang X. Jiang H. Wang Y. (2015). Decreased diversity of the oral microbiota of patients with Hepatitis B virus-induced chronic liver disease, a pilot project. *Sci. Rep.* 5:17098 10.1038/srep17098 PMC4660595 26606973

[B44] Lips K. R. Brem F. Brenes R. Reeve J. D. Alford R. A. Voyles J. (2006). Emerging infectious disease and the loss of biodiversity in a Neotropical amphibian community. *Proc. Natl. Acad. Sci. U.S.A.* 103 3165–3170. 10.1073/pnas.0506889103 16481617 PMC1413869

[B45] Lips K. R. Diffendorfer J. Mendelson J. R. III Sears M. W. (2008). Riding the wave: reconciling the roles of disease and climate change in amphibian declines. *PLoS Biol.* 6:e72 10.1371/journal.pbio.0060072 PMC2270328 18366257

[B46] Lokmer A. Wegner K. M. (2015). Hemolymph microbiome of Pacific oysters in response to temperature, temperature stress and infection. *ISME J.* 9 670–682. 10.1038/ismej.2014.160 25180968 PMC4331581

[B47] Longo A. V. Savage A. E. Hewson I. Zamudio K. R. (2015). Seasonal and ontogenetic variation of skin microbial communities and relationships to natural disease dynamics in declining amphibians. *R. Soc. Open Sci.* 2:140377 10.1098/rsos.140377 PMC4632566 26587253

[B48] Longo A. V. Zamudio K. R. (2017). Environmental fluctuations and host skin bacteria shift survival advantage between frogs and their fungal pathogen. *ISME J.* 11 349–361. 10.1038/ismej.2016.138 27935596 PMC5270579

[B49] Madison J. D. Berg E. A. Abarca J. G. Whitfield S. M. Gorbatenko O. Pinto A. (2017). Characterization of *Batrachochytrium dendrobatidis* inhibiting bacteria from amphibian populations in Costa Rica. *Front. Microbiol.* 8:290 10.3389/fmicb.2017.00290 PMC5329008 28293222

[B50] Martel A. Blooi M. Adriaensen C. Van Rooij P. Beukema W. Fisher M. C. (2014). Recent introduction of a chytrid fungus endangers Western Palearctic salamanders. *Science* 346 630–631. 10.1073/pnas.1307356110 25359973 PMC5769814

[B51] Martel A. Spitzen-van der Sluijs A. Blooi M. Bert W. Ducatelle R. Fisher M. C. (2013). *Batrachochytrium salamandrivorans* sp nov causes lethal chytridiomycosis in amphibians. *Proc. Natl. Acad. Sci. U.S.A.* 110 15325–15329. 10.1126/science.1258268 24003137 PMC3780879

[B52] Muletz C. R. Myers J. M. Domangue R. J. Herrick J. B. Harris R. N. (2012). Soil bioaugmentation with amphibian cutaneous bacteria protects amphibian hosts from infection by *Batrachochytrium dendrobatidis*. *Biol. Conserv.* 152 119–126. 10.1016/j.biocon.2012.03.002

[B53] Muletz-Wolz C. R. Direnzo G. V. Yarwood S. A. Campbell Grant E. H. Fleischer R. C. Lips K. R. (2017a). Antifungal bacteria on woodland salamander skin exhibit high taxonomic diversity and geographic variability. *Appl. Environ. Microbiol.* 83:e00186–17. 10.1128/AEM.00186-17 28213545 PMC5394319

[B54] Muletz-Wolz C. R. Yarwood S. A. Campbell Grant E. H. Fleischer R. C. Lips K. R. (2017b). Effects of host species and environment on the skin microbiome of Plethodontid salamanders. *J. Anim. Ecol.* 10.1111/1365-2656.12726 [Epub ahead of print]. 28682480

[B55] Nebergall E. E. (2013). *Ecology and Applications of Cutaneous Mechanisms of Resistance to Amphibian Chytridiomycosis.* Master’s thesis, Oregon State University Corvallis, OR.

[B56] Piotrowski J. S. Annis S. L. Longcore J. E. (2004). Physiology of *Batrachochytrium dendrobatidis*, a chytrid pathogen of amphibians. *Mycologia* 96 9–15. 10.2307/3761981 21148822

[B57] R Core Team (2016). *R: A Language and Environment for Statistical Computing.* Vienna: R Foundation for Statistical Computing Available at: https://www.R-project.org/

[B58] Raaijmakers J. M. Vlami M. de Souza J. T. (2002). Antibiotic production by bacterial biocontrol agents. *Antonie Van Leeuwenhoek* 81 537–547. 10.1023/a:1020501420831 12448749

[B59] Ravel J. Wellington E. M. H. Hill R. T. (2000). Interspecific transfer of *Streptomyces* giant linear plasmids in sterile amended soil microcosms. *Appl. Environ. Microbiol.* 66 529–534. 10.1128/AEM.66.2.529-534.2000 10653714 PMC91859

[B60] Ribas L. Li M. -S. Doddington B. J. Robert J. Seidel J. A. Kroll J. S. (2009). Expression profiling the temperature-dependent amphibian response to infection by *Batrachochytrium dendrobatidis*. *PLoS ONE* 4:e8408 10.1371/journal.pone.0008408 PMC2794374 20027316

[B61] Rosenblum E. B. James T. Y. Zamudio K. R. Poorten T. J. Ilut D. Rodriguez D. (2013). Complex history of the amphibian-killing chytrid fungus revealed with genome resequencing data. *Proc. Natl. Acad. Sci. U.S.A.* 110 9385–9390. 10.1073/pnas.1300130110 23650365 PMC3677446

[B62] Rovenich H. Boshoven J. C. Thomma B. (2014). Filamentous pathogen effector functions, of pathogens, hosts and microbiomes. *Curr. Opin. Plant Biol.* 20 96–103. 10.1016/j.pbi.2014.05.001 24879450

[B63] Schloegel L. M. Toledo L. F. Longcore J. E. Greenspan S. E. Vieira C. A. Lee M. (2012). Novel, panzootic and hybrid genotypes of amphibian chytridiomycosis associated with the bullfrog trade. *Mol. Ecol.* 21 5162–5177. 10.1111/j.1365-294X.2012.05710.x 22857789

[B64] Spitzen-van der Sluijs A. Martel A. Asselberghs J. Bales E. K. Beukema W. Bletz M. C. (2016). Expanding distribution of lethal amphibian fungus *Batrachochytrium salamandrivorans* in Europe. *Emerg. Infect. Dis.* 22 1286–1288. 10.3201/eid2207.160109 27070102 PMC4918153

[B65] Stegen G. Pasmas F. Schmidt B. R. Rouffaer L. O. Van Praet S. Schaub M. (2017). Drivers of salamander extirpation mediated by *Batrachochytrium salamandrivorans*. *Nature* 544 353–356. 10.1038/nature22059 28425998

[B66] Stevenson L. A. Alford R. A. Bell S. C. Roznik E. A. Berger L. Pike D. A. (2013). Variation in thermal performance of a widespread pathogen, the amphibian chytrid fungus *Batrachochytrium dendrobatidis*. *PLoS ONE* 8:e73830 10.1371/journal.pone.0073830 PMC3762749 24023908

[B67] Suzuki R. Shimodaira H. (2006). Pvclust, an R package for assessing the uncertainty in hierarchical clustering. *Bioinformatics* 22 1540–1542. 10.1093/bioinformatics/btl117 16595560

[B68] Teplitski M. Ritchie K. (2009). How feasible is the biological control of coral diseases? *Trends Ecol. Evol.* 24 378–385. 10.1016/j.tree.2009.02.008 19406502

[B69] Verschuere L. Rombaut G. Sorgeloos P. Verstraete W. (2000). Probiotic bacteria as biological control agents in aquaculture. *Microbiol. Mol. Biol. Rev.* 64 655–671. 10.1128/mmbr.64.4.655-671.2000 11104813 PMC99008

[B70] Woodhams D. C. Alford R. A. Antwis R. E. Archer H. Becker M. H. Belden L. K. (2015). Antifungal isolates database of amphibian skin-associated bacteria and function against emerging fungal pathogens. *Ecology* 96 595–595. 10.1890/14-1837.1

[B71] Woodhams D. C. Alford R. A. Briggs C. J. Johnson M. Rollins-Smith L. A. (2008). Life-history trade-offs influence disease in changing climates, Strategies of an amphibian pathogen. *Ecology* 89 1627–1639. 10.1890/06-1842.1 18589527

[B72] Woodhams D. C. Brandt H. Baumgartner S. Kielgast J. Kuepfer E. Tobler U. (2014). Interacting symbionts and immunity in the amphibian skin mucosome predict disease risk and probiotic effectiveness. *PLoS ONE* 9:e96375 10.1371/journal.pone.0096375 PMC4005770 24789229

[B73] Woodhams D. C. Geiger C. C. Reinert L. K. Rollins-Smith L. A. Lam B. Harris R. N. (2012). Treatment of amphibians infected with chytrid fungus, learning from failed trials with itraconazole, antimicrobial peptides, bacteria, and heat therapy. *Dis. Aquat. Organ.* 98 11–25. 10.3354/dao02429 22422126

[B74] Xue C. Penton C. R. Shen Z. Z. Zhang R. F. Huang Q. W. Li R. (2015). Manipulating the banana rhizosphere microbiome for biological control of Panama disease. *Sci. Rep.* 5 14596 10.1038/srep11124 PMC4525139 26242751

[B75] Yasumiba K. Bell S. Alford R. (2016). Cell density effects of frog skin bacteria on their capacity to inhibit growth of the chytrid fungus, *Batrachochytrium dendrobatidis*. *Microb. Ecol.* 71 124–130. 10.1007/s00248-015-0701-9 26563320

