# Supplementary material for: Inhibition of Fungal Pathogens across Genotypes and Temperatures by Amphibian Skin Bacteria
Source: Front Microbiol. 2017 Aug 21;8:1551. doi: 10.3389/fmicb.2017.01551 (PMC5566582; doi:10.3389/fmicb.2017.01551)
Supplement: Supplementary file 1 [file Data_Sheet_1.DOCX]

**Supporting information for:**

**‘Amphibian skin bacteria inhibit fungal pathogens across genotypes and temperatures’**

Carly R. Muletz-Wolz, Jose G. Almario, Samuel E. Barnett, Graziella V. DiRenzo, An Martel, Frank Pasmans, Kelly R. Zamudio, L. Felipe Toledo, Karen R. Lips

*To whom correspondence should be addressed. E-mail: craemuletz@gmail.com

This file includes:

Figure S1. Set-up for 96-well plates

Figure S2. Incubator set-up

Figure S3. Comparison of inhibition scores among bacterial and control wells

Table S1. Mean inhibition score for each bacteria-temperature-genotype combination

Table S2. Post-hoc analyses for bacterial strains displaying a temperature main effect

Table S3. Post-hoc analyses for bacterial strains displaying a genotype main effect

**Figure S1.** Set-up for 96-well plates. We randomly generated four configurations of bacterial by *Batrachochytrium* combinations. One plate of each configuration was housed within an incubator.

**Figure S2.** Incubator set-up. We randomly assigned two incubators to one of two temperatures (12 or 18 °C), and maintained them at that temperature, a 12 h light, 12 h dark cycle and 80 ± 10% humidity throughout the experiment. We randomly assigned four plates to an incubator such that each incubator housed all plate configurations, with all plates on the same shelf within an incubator.

**Figure S3.** Comparison of inhibition scores among bacterial and control wells for 12 ^°^C (a) and 18 ^°^C (b). Inhibition scores were calculated based on sample slope of growth compared to nutrient-depleted positive control (NDPC) slope of growth. No bacteria were considered facultative of *Batrachochytrium* growth, as all inhibition scores were significantly greater than the respective inhibition score for the positive control (PC) wells.

**Table S1.** Mean inhibition score ± standard deviation for each bacteria-temperature-genotype combination, including samples sizes (N). Scores indicating inhibition are highlighted in gray.

| **Bacteria** | **Temp** | ***Batrachochytrium* genotype** | **N** | **Mean inhibition score ± SD (%)** |
| --- | --- | --- | --- | --- |
| *A. rhizosphaerae* | 12 | Bsal | 12 | 46 ± 33 |
| *A. rhizosphaerae* | 12 | BdGPL1-JEL404 | 16 | -37 ± 11 |
| *A. rhizosphaerae* | 12 | BdGPL2-JEL423 | 13 | -67 ± 26 |
| *A. rhizosphaerae* | 12 | BdGPL1-JEL647 | 16 | -52 ± 34 |
| *A. rhizosphaerae* | 12 | BdBrazil-JEL649 | 10 | -48 ± 55 |
| *A. rhizosphaerae* | 12 | BdGPL2-SRS810 | 16 | -80 ± 36 |
| *A. rhizosphaerae* | 18 | Bsal | 14 | 53 ± 25 |
| *A. rhizosphaerae* | 18 | BdGPL1-JEL404 | 14 | -75 ± 72 |
| *A. rhizosphaerae* | 18 | BdGPL2-JEL423 | 16 | -33 ± 20 |
| *A. rhizosphaerae* | 18 | BdGPL1-JEL647 | 16 | -63 ± 41 |
| *A. rhizosphaerae* | 18 | BdBrazil-JEL649 | 15 | -36 ± 34 |
| *A. rhizosphaerae* | 18 | BdGPL2-SRS810 | 15 | -61 ± 48 |
| *Pseudomonas* sp. strain 1 | 12 | Bsal | 16 | 65 ± 13 |
| *Pseudomonas* sp. strain 1 | 12 | BdGPL1-JEL404 | 16 | 37 ± 12 |
| *Pseudomonas* sp. strain 1 | 12 | BdGPL2-JEL423 | 16 | 11 ± 20 |
| *Pseudomonas* sp. strain 1 | 12 | BdGPL1-JEL647 | 16 | 42 ± 12 |
| *Pseudomonas* sp. strain 1 | 12 | BdBrazil-JEL649 | 10 | 119 ± 4 |
| *Pseudomonas* sp. strain 1 | 12 | BdGPL2-SRS810 | 16 | 29 ± 49 |
| *Pseudomonas* sp. strain 1 | 18 | Bsal | 16 | 47 ± 31 |
| *Pseudomonas* sp. strain 1 | 18 | BdGPL1-JEL404 | 16 | 0 ± 39 |
| *Pseudomonas* sp. strain 1 | 18 | BdGPL2-JEL423 | 16 | 25 ± 9 |
| *Pseudomonas* sp. strain 1 | 18 | BdGPL1-JEL647 | 16 | 5 ± 15 |
| *Pseudomonas* sp. strain 1 | 18 | BdBrazil-JEL649 | 11 | 104 ± 5 |
| *Pseudomonas* sp. strain 1 | 18 | BdGPL2-SRS810 | 16 | -51 ± 33 |
| *Pseudomonas* sp. strain 2 | 12 | Bsal | 15 | 92 ± 3 |
| *Pseudomonas* sp. strain 2 | 12 | BdGPL1-JEL404 | 13 | 98 ± 1 |
| *Pseudomonas* sp. strain 2 | 12 | BdGPL2-JEL423 | 15 | 87 ± 5 |
| *Pseudomonas* sp. strain 2 | 12 | BdGPL1-JEL647 | 16 | 62 ± 14 |
| *Pseudomonas* sp. strain 2 | 12 | BdBrazil-JEL649 | 12 | 57 ± 27 |
| *Pseudomonas* sp. strain 2 | 12 | BdGPL2-SRS810 | 14 | 95 ± 3 |
| *Pseudomonas* sp. strain 2 | 18 | Bsal | 16 | 92 ± 6 |
| *Pseudomonas* sp. strain 2 | 18 | BdGPL1-JEL404 | 16 | 93 ± 5 |
| *Pseudomonas* sp. strain 2 | 18 | BdGPL2-JEL423 | 16 | 94 ± 3 |
| *Pseudomonas* sp. strain 2 | 18 | BdGPL1-JEL647 | 14 | 83 ± 7 |
| *Pseudomonas* sp. strain 2 | 18 | BdBrazil-JEL649 | 13 | 92 ± 4 |
| *Pseudomonas* sp. strain 2 | 18 | BdGPL2-SRS810 | 13 | 96 ± 4 |
| *Chyrseobacterium* sp. | 12 | Bsal | 16 | 71 ± 19 |
| *Chyrseobacterium* sp. | 12 | BdGPL1-JEL404 | 16 | 4 ± 34 |
| *Chyrseobacterium* sp. | 12 | BdGPL2-JEL423 | 16 | -87 ± 39 |
| *Chyrseobacterium* sp. | 12 | JBdGPL1-JEL647 | 16 | -33 ± 24 |
| *Chyrseobacterium* sp. | 12 | BdBrazil-JEL649 | 11 | 38 ± 40 |
| *Chyrseobacterium* sp. | 12 | BdGPL2-SRS810 | 16 | -84 ± 37 |
| *Chyrseobacterium* sp. | 18 | Bsal | 16 | 43 ± 40 |
| *Chyrseobacterium* sp. | 18 | BdGPL1-JEL404 | 16 | -56 ± 41 |
| *Chyrseobacterium* sp. | 18 | BdGPL2-JEL423 | 16 | -36 ± 22 |
| *Chyrseobacterium* sp. | 18 | BdGPL1-JEL647 | 16 | 5 ± 21 |
| *Chyrseobacterium* sp. | 18 | BdBrazil-JEL649 | 16 | 43 ± 28 |
| *Chyrseobacterium* sp. | 18 | BdGPL2-SRS810 | 16 | -109 ± 39 |
| *Stenotrophomonas* sp. | 12 | Bsal | 16 | 92 ± 4 |
| *Stenotrophomonas* sp. | 12 | BdGPL1-JEL404 | 15 | 100 ± 3 |
| *Stenotrophomonas* sp. | 12 | BdGPL2-JEL423 | 13 | 88 ± 12 |
| *Stenotrophomonas* sp. | 12 | BdGPL1-JEL647 | 9 | 71 ± 26 |
| *Stenotrophomonas* sp. | 12 | BdBrazil-JEL649 | 9 | 70 ± 31 |
| *Stenotrophomonas* sp. | 12 | BdGPL2-SRS810 | 14 | 98 ± 6 |
| *Stenotrophomonas* sp. | 18 | Bsal | 12 | 91 ± 6 |
| *Stenotrophomonas* sp. | 18 | BdGPL1-JEL404 | 11 | 96 ± 1 |
| *Stenotrophomonas* sp. | 18 | BdGPL2-JEL423 | 14 | 96 ± 7 |
| *Stenotrophomonas* sp. | 18 | BdGPL1-JEL647 | 12 | 96 ± 13 |
| *Stenotrophomonas* sp. | 18 | BdBrazil-JEL649 | 15 | 100 ± 5 |
| *Stenotrophomonas* sp. | 18 | BdGPL2-SRS810 | 13 | 100 ± 7 |
|  |  | **TOTAL:** | 867 |  |

Table S2. Post-hoc analyses for bacterial strains that displayed a significant main effect for temperature. *Chryseobacterium* sp. and *A. rhizosphaerae* did not show differences in inhibition within *Batrachochytrium* genotypes between temperatures.

|  | *Stenotrophomonas* sp. | *Pseudomonas* sp. strain 1 | *Pseudomonas* sp. strain 2 |
| --- | --- | --- | --- |
| Bsal | ns | ns | ns |
| BdGPL1-JEL404 | ns | 0.0071 | ns |
| BdGPL2-JEL423 | ns | ns | ns |
| BdGPL1-JEL647 | 0.0035 | 0.0068 | 0.0008 |
| BdBrazil-JEL649 | 0.0007 | ns | < 0.0001 |
| BdGPL2-SRS810 | ns | < 0.0001 | ns |

Table S3. Post-hoc analyses for bacterial strains that displayed a significant main effect for pathogen genotype.

| **12 °C** | | | | | |
| --- | --- | --- | --- | --- | --- |
|  | *A. rhizosphaerae* | *Pseudomonas* sp. strain 1 | *Pseudomonas* sp. strain 2 | *Chryseobacterium* sp. | *Stenotrophomonas* sp. |
| Bsal/ GPL1-JEL404 | <0.0001 | 0.0061 | ns | ns | ns |
| Bsal/ GPL1-JEL423 | <0.0001 | <0.0001 | ns | ns | ns |
| Bsal/ GPL1-JEL647 | 0.0008 | ns | 0.0001 | 0.0002 | ns |
| Bsal/ Brazil-JEL649 | 0.0116 | 0.0009 | <0.0001 | ns | ns |
| Bsal/ GPL1-SRS810 | <0.0001 | ns | ns | ns | ns |
| GPL1-JEL404/ GPL1-JEL423 | ns | 0.0129 | 0.0007 | ns | 0.0162 |
| GPL1-JEL404/ GPL1-JEL647 | ns | ns | <0.0001 | ns | 0.0061 |
| GPL1-JEL404/ Brazil-JEL649 | ns | <0.0001 | <0.0001 | ns | 0.0055 |
| GPL1-JEL404/ GPL1-SRS810 | ns | ns | ns | 0.0017 | ns |
| GPL1-JEL423/ GPL1-JEL647 | ns | ns | 0.0009 | ns | ns |
| GPL1-JEL423/ Brazil-JEL649 | ns | <0.0001 | 0.0001 | ns | ns |
| GPL1-JEL423/ GPL1-SRS810 | ns | ns | ns | ns | ns |
| GPL1-JEL647/ Brazil-JEL649 | ns | <0.0001 | ns | <0.0001 | ns |
| GPL1-JEL647/ GPL1-SRS810 | ns | ns | <0.0001 | <0.0001 | <0.0001 |
| GPL1-SRS810/ Brazil-JEL649 | 0.0098 | <0.0001 | <0.0001 | <0.0001 | <0.0001 |
|  |  |  |  |  |  |
| **18 °C** | | | | | |
|  | *A. rhizosphaerae* | *Pseudomonas* sp. strain 1 | *Pseudomonas* sp. strain 2 | *Chryseobacterium* sp. | *Stenotrophomonas* sp. |
| Bsal/ GPL1-JEL404 | <0.0001 | <0.0001 | ns | <0.0001 | ns |
| Bsal/ GPL1-JEL423 | <0.0001 | 0.0434 | ns | <0.0001 | ns |
| Bsal/ GPL1-JEL647 | 0.0001 | 0.0183 | ns | ns | ns |
| Bsal/ Brazil-JEL649 | 0.0047 | 0.0019 | ns | ns | ns |
| Bsal/ GPL1-SRS810 | 0.0002 | <0.0001 | ns | <0.0001 | ns |
| GPL1-JEL404/ GPL1-JEL423 | 0.0026 | 0.0276 | ns | ns | ns |
| GPL1-JEL404/ GPL1-JEL647 | ns | ns | ns | 0.0468 | ns |
| GPL1-JEL404/ Brazil-JEL649 | ns | <0.0001 | ns | 0.0004 | ns |
| GPL1-JEL404/ GPL1-SRS810 | ns | 0.0026 | ns | ns | ns |
| GPL1-JEL423/ GPL1-JEL647 | ns | ns | ns | ns | ns |
| GPL1-JEL423/ Brazil-JEL649 | ns | <0.0001 | ns | 0.0057 | ns |
| GPL1-JEL423/ GPL1-SRS810 | ns | <0.0001 | ns | 0.0109 | ns |
| GPL1-JEL647/ Brazil-JEL649 | ns | <0.0001 | 0.015 | 0.0003 | ns |
| GPL1-JEL647/ GPL1-SRS810 | ns | <0.0001 | 0.0001 | <0.0001 | ns |
| GPL1-SRS810/ Brazil-JEL649 | ns | <0.0001 | ns | <0.0001 | ns |
